# IBD and Bile Acid Absorption: Focus on Pre-clinical and Clinical Observations

**DOI:** 10.3389/fphys.2020.00564

**Published:** 2020-06-12

**Authors:** Leo R. Fitzpatrick, Paniz Jenabzadeh

**Affiliations:** Department of Pharmaceutical and Biomedical Sciences, College of Pharmacy, California Northstate University, Elk Grove, CA, United States

**Keywords:** bile acid malabsorption, inflammatory bowel disease, colitis, therapeutic options, Crohn’s disease, diarrhea, animal models, bile

## Abstract

Inflammatory bowel disease (IBD) causes chronic inflammation affecting the GI tract. It is classified as consisting of Crohn’s Disease (CD) and Ulcerative Colitis (UC). Bile Acid absorption is altered in both pre-clinical models of Inflammatory Bowel Disease (IB) and in human IBD. The bile acid transporter apical sodium dependent bile acid transporter (ASBT) showed decreased expression in rats with TNBS colitis. Decreased ASBT expression has also been described in murine, canine and rabbit models of intestinal inflammation. Human IBD studies have shown that an inflamed ileum can interrupt enterohepatic recirculation of bile acid, which could be due to inflammatory cytokine induced repression of the ASBT promoter. There are different hypotheses as to why ASBT is downregulated during CD. In addition, one study has demonstrated the beneficial effect of a glucocorticoid on ASBT expression, when treating IBD. Our aim in this paper was to systematically review various aspects of bile acid malabsorption in animal models of intestinal inflammation, as well as in IBD.

## Bile Acid Synthesis and Absorption in Humans and Rodents

Bile acid, the end product of cholesterol catabolism, is synthesized in the liver through multiple oxidation and hydroxylation mechanisms (Dietschy, 1968; Dawson and Karpen, 2015). In humans, the bile acids synthesized in the liver are referred to as primary bile acid, including cholic acid (CA) and chenodeoxycholic acid (CDCA) (Dawson and Karpen, 2015). The secondary bile acids are the transformed form of primary bile acid carried out by gut bacteria through a variety of reactions such as dihydroxylation, dehydrogenation, and epimerization (Dawson and Karpen, 2015). The secondary bile acids include deoxycholic acid (DCA), ursocholic acid (UCA), ursodeoxycholic acid (UDCA), and lithocholic acid (LCA) (Dawson and Karpen, 2015).

Bile salts (i.e., conjugated bile acids) exist in different polarities that leads to having at least three different physiological routes of intestinal absorption including: (1) the jejunal passive route, (2) the ileal active and passive transport, and (3) the colonic passive route (Madsen, 1990). Several studies have indicated that the major route of absorption for most conjugated bile acids is the ileal active transport route, while the passive absorption present throughout the small intestine is the main route for unconjugated and some glycine-conjugated bile acids (Dawson and Karpen, 2015). The design of bile acid transport is so efficient that only 5% of bile acid in the lumen is excreted in feces and the other 95% returns to the liver through the enterohepatic system (Martínez-Augustin and de Medina, 2008; Dawson and Karpen, 2015). Intuitively, these processes make sense as bile acids become more hydrophobic through deconjugation process by gut bacteria allowing for passive transport. In this regard, there is also decreased expression of the apical sodium dependent bile acid transporter (ASBT) in the colon (Madsen, 1990; Hruz et al., 2006).

There are various studies indicating that ASBT is the major transporter responsible for efficient uptake of bile acid in the terminal ileum (Dawson and Karpen, 2015). The location of this transporter has been shown in diagrammatic form in previous publications (e.g., Jahnel et al., 2014).

The inactivation of ASBT gene in mice caused a reduction in the enterohepatic circulation of bile acids (Dawson et al., 2003; Jung et al., 2007; Vivian et al., 2014). Also, there is evidence showing intestinal bile acid malabsorption (BAM) in humans with an ASBT loss-of-function mutation (Oelkers et al., 1997). Of pertinence to this review, the first report of a dysfunctional ASABT mutation was found in the ileum of a Crohn’s Disease (CD) patient (Wong et al., 1995; Kwon and Carey, 2004). Additionally, reduced bile acid absorption occurs in animal models after administration of small molecule inhibitors of the ASBT (Huang et al., 2005; Wu et al., 2013).

There is an assumption that there must be a cytosolic protein aiding with bile acid transport across the enterocyte just as bile acid is bound to other cytosolic proteins including albumin in plasma (Rudman and Kendall, 1957; Ceryak et al., 1993; Kramer, 1995). It has been found that this protein is the ileal bile acid-binding protein (IBABP), which is an abundant cytosolic protein (Smathers and Petersen, 2011). Finally, the transported bile acid exits the enterocyte via organic solute transporter (OSTα/β transporter), functioning in a sodium-independent manner, and predominantly enters the portal venous system (Dietschy, 1968; Martínez-Augustin and de Medina, 2008).

In a pre-clinical model, Farnesoid X Receptor (FXR) activation causes reduced bile acid uptake by downregulation of ASBT expression in enterocytes. Moreover, FXR activation promotes the excretion of bile acids on the basolateral side of the enterocyte by increasing OSTα and OSTβ expression, which affects the transport of bile acids into the circulation (Tiratterra et al., 2018).

It should also be noted that there are differences in bile acid metabolism between humans and rodents (mice, rats). For example, in rodents bile acids are hydroxylated at the 6-beta position to form muricholoic acids (MCAs), which is a group of bile acids unique to these species (Rudling, 2016).

Bile acid absorption in the terminal ileum (and colon) is a critical component for their enterohepatic circulation. This process can be altered during Inflammatory Bowel Disease (IBD). In this regard, diarrhea in patients with IBD may be partially dependent on bile acid malabsorption (Jahnel et al., 2014).

## Pre-clinical Observations

Pertinent information is presented below from rodent, rabbit, canine and non-human primate models, as related to this overall topic. Moreover, pre-clinical observations related to bile acid transport proteins (e.g., ASBT) and fecal bile acids in animal models of IBD/intestinal inflammation are summarized in Table 1.

**TABLE 1:** Bile acid transport proteins and fecal bile acids in animal models of IBD/Intestinal inflammation.

IBD model/species	Reference number	Effect(s) on BA transporters	Effect(s) on bile acids or lipids
TNBS/Rat	21	Decreased ASBT expression (60% during acute phase).	Total bile acid levels in the feces were increased 1.6 fold.
TNBS/Hamster	25	Not measured	Decreased bile acid uptake in the ileum
TNF^ΔARE/WT^/ Mouse	26	Decreased ASBT expression (22%). Also decreased expression of Ostα and Ostβ transporters.	Not measured
IL-10 deficient Mouse	28	Increased ASBT expression	Decreased total fecal bile acids
DSS/Mouse	31	Not measured	Increased fecal lipids in mice with colitis.
DSS/Rat	35	Not measured	Increased fecal cholic acid
Chronic Inflammatory Enteropathy/Dog	36	Decreased ASBT expression	Increased fecal primary bile acids
Chronic Enteropathy/Dog	38	Not measured	Decreased fecal secondary bile acids
Indomethacin/Mouse	41	Decreased ASBT, decreased Ostα and Ostβ	Increased fecal bile acid excretion (32%)
Indomethacin/Rat	42	Decreased mRNA and protein levels of ASBT	Not measured
Ileitis/Rabbit	41,43	Decreased mRNA and protein levels of ASBT	Decreased uptake of bile acid

The intracolonic administration of Trinitrobenzene Sulfonic Acid (TNBS) to rodents (rats, mice) is a commonly used model of CD (Te Velde et al., 2006; Fitzpatrick et al., 2012; Hou et al., 2018; Fitzpatrick et al., 2020). Using this model, investigators showed decreased expression of intestinal ASBT primarily during the acute phase of the colitis (Hou et al., 2018). Of note, in conjunction with the decreased expression of ASBT, there was increased fecal excretion of bile acids, as well as diarrhea in rats with acute TNBS-induced colitis (Jahnel et al., 2014). The diarrhea was likely due to the excess bile acids in the colon, which would stimulate increased secretion of electrolytes/water, as well as increased motility (Hou et al., 2018). Interestingly, in conjunction with this study, intestinal FGR expression was not affected, but expression of a target gene [small heterodimer partner (SHP)] was significantly reduced (Jahnel et al., 2014). Using a variation of the standard model, investigators showed that instillation of TNBS into the ileum of hamsters resulted in ileitis. In animals with ileitis, active bile acid uptake (i.e., taurocholate) decreased by 84% in the terminal ileum and by 58% in the proximal ileum (Steizner et al., 2001).

The TNF^ΔARE/WT^ murine model of ileitis is a model of CD that is dependent on the pro-inflammatory cytokine TNF-α (Kontoyiannis et al., 1999; van den Bossche et al., 2017). With this model, investigators recently showed decreased expression of ASBT, as well as the basolateral enterocyte efflux transporters, Organic Solute Transporters alpha and beta (Ostα and Ostβ) in mice with ileitis (van den Bossche et al., 2017). Interestingly, administration of tauroursodeoxycholic acid (TUDCA) (in the drinking water) to mice partially reversed the attenuated expression of these transport proteins. Moreover, TUDCA partially improved histopathological and “clinical” parameters of murine ileitis (van den Bossche et al., 2017). The investigators emphasized that this ileitis model was a more appropriate animal model of IBD to study alterations in bile acid transporters, because >95% of the intestinal bile acid pool does not transit to the colon due to typically efficient reabsorption in the distal ileum (van den Bossche et al., 2017).

IL-10 deficient mice develop a chronic inflammation in the intestinal tract, including the colon and ileum (Kühn et al., 1993; Rau et al., 2016). Using this CD model, intriguing results were reported by Rau and colleagues (Rau et al., 2016). They found increased ileal ASBT expression in mice with colitis (albeit at the mRNA level). This seemed to coincide with a decreased total fecal bile acid content in mice with colitis. Is it possible that due to the chronic nature of the intestinal inflammation (i.e., mice were euthanized at 6–12 months of age) that there was compensatory rebound effect involving the ASBT bile acid transporter, and consequently ileal uptake of bile acids?

Dextran Sulfate Sodium (DSS) administered in the drinking water is a common way of inducing colitis in mice (Fitzpatrick et al., 2014; Bitzer et al., 2016). Using this model of Ulcerative Colitis (UC), Bitzer et al. demonstrated a significant increase in total fecal lipids within the colons of mice with colitis. These results indicate overall lipid malabsorption in conjunction with this colitis model. Unfortunately, intestinal bile acid levels were not measured with this study (Bitzer et al., 2016).

DCA is hydrophobic. Therefore, it can cause cytotoxicity to cells. Some investigators have shown that higher fecal bile acid hydrophobicity is associated with an exacerbation of DSS-induced colitis in mice (Stenman et al., 2013). Moreover, Zhao et al. showed that intracolonic administration of DCA worsened murine DSS-induced colitis. This pro-colitis effect was associated with an increased colonic IL-1β content, in conjunction with NLRP3 inflammasome activation. From a potential therapeutic standpoint, treatment with a caspase 1 inhibitor (Belnacasan) improved parameters of colitis (Zhao et al., 2016).

Recently, the same group of investigators suggested that exacerbation of DSS-induced colitis in mice by DCA involved the sphingosine-1-phosphate 2 receptor (SIPR2). Treatment with a S1PR2 antagonist improved this colitis. Therefore, targeting the S1PR2 may represent a rationale pharmacological approach for intestinal inflammation, in certain patients still consuming a high fat diet (Zhao et al., 2018). Araki et al. found significantly increased cholic acid levels in rats with DSS-induced colitis. Interestingly, there was a significant association between the concentration of fecal cholic acid and area of macroscopic injury in the rat colon (Araki et al., 2001).

Dogs with chronic inflammatory enteropathy (CIE) have evidence of intestinal inflammation, fecal bile acid dysmetabolism and persistent diarrhea (Giaretta et al., 2018; Guard et al., 2019). Giaretta and colleagues found that dogs with CIE had decreased ileal ASBT protein expression. Interestingly, there was a significant negative correlation in this study between the cumulative histopathology score in the ileum and ileal ASBT expression. Moreover, dogs with CIE also had increased primary bile acids (e.g., chenodeoxycholic acid) in the feces compared to the control group of animals, suggesting bile acid dysmetabolism (Giaretta et al., 2018). Moreover, secondary fecal unconjugated bile aids were decreased in dogs with a steroid responsive form of IE. Upon treatment of dogs with prednisone, there was a drug-induced increase in the fecal unconjugated bile content. It was concluded that corticosteroids therapeutically managed canine CIE by affecting bile acid dysmetabolism (Guard et al., 2019).

A very recent publication revealed that dogs classified as having Chronic Enteropathy (CE) showed reduced secondary bile acids (lithocholic and deoxycholic acid) in the feces, accompanied by intestinal microbial dysbiosis (Wang et al., 2019). Treatment with a hydrolyzed protein diet decreased the abundance of pathogenic bacterial species (e.g., *Escherichia coli* and *Clostridium perfringens*), and concomitantly increased the levels of secondary bile acids. Of note, there was a quick and prolonged clinical response to the diet-based therapy in most dogs with CE. Interestingly, the investigators found that levels of a bile acid producing bacteria (*Clostridium hiranonis*) were increased after dietary therapy and was linked to the clinical remission found in dogs with CE. Finally, when *Clostridium hiranonis* was administered to mice with DSS-induced colitis there was an amelioration of disease activity (Wang et al., 2019).

Cotton top tamarins are small “New World Monkeys,” which have a propensity to spontaneously develop idiopathic colitis and colonic adenocarcinoma (Ausman et al., 1993; Watkins et al., 1997). It is recognized as a unique non-human primate model of UC (Watkins et al., 1997). Ausman and colleagues conducted a study that monitored fecal bile acids in these animals. They observed lower levels of secondary bile acids (indicating a reduced rate of microbial cholesterol conversion) in cotton top tamarins compared to humans and other animal species (Ausman et al., 1993).

Indomethacin-induced acute ileitis led to repression of ASBT in wild-type mice. In contrast, activation of ASBT was seen in c-fos–null mice. Counter-intuitively, indomethacin-induced ileal damage was greater in these c-fos–null mice compared with wild-type mice. Indomethacin treatment also led to repression of both Ostα and Ostβ mRNAs. Fecal bile acid excretion was also increased by 32% in mice treated with indomethacin. Mechanistically, the investigators concluded that mouse ASBT is inhibited by inflammatory cytokines via direct interactions of c-fos with the ASBT promoter (Neimark et al., 2006). In turn, this impacts fecal bile acid levels in mice. They also showed that Indomethacin-induced ileitis in Lewis rats resulted in specific reductions in ileal ASBT messenger RNA and protein levels, whereas c-jun and c-fos proteins were induced in the study. The authors suggested that such “inflammation is associated with up-regulation, phosphorylation, and nuclear translocation of c-fos, which then represses ASBT promoter activity via binding of the 3’ AP-1 element by a c-fos/c-jun heterodimer” (Chen et al., 2002).

A rabbit model of ileitis was also reported to be associated with the downregulation of ASBT, at both the messenger RNA and protein levels (Sundaram et al., 1998; Neimark et al., 2006). Moreover, designed kinetic studies demonstrated that sodium-bile acid cotransport was inhibited by a decrease in both the affinity and maximal rate of uptake, of bile acids (Sundaram et al., 1998).

Finally, using a murine colitis associated cancer (CAC) model; investigators showed decreased intestinal mRNA levels of ASBT. Additionally, primary bile acids and taurine-conjugates were increased in the feces of CAC mice (Cao et al., 2016). Of note, ileal FXR expression was significantly reduced in CAC mice, and proposed to play a prominent role in the carcinogenesis process (Cao et al., 2016).

It should be emphasized that a common mechanism involved in these preclinical models of IBD/intestinal inflammation is decreased ASBT expression (Sundaram et al., 1998; Chen et al., 2002; Neimark et al., 2006; van den Bossche et al., 2017; Giaretta et al., 2018; Hou et al., 2018). This information is summarized within Table 1.

## Clinical Observations

In order to confirm the bile acid malabsorption in Crohn’s disease (CD) patients, Nishida and colleagues administered chenodeoxycholic-11, 12-d_2_ acid to eight patients and compared them with four volunteer control patients (Nishida et al., 1982). The results of this study demonstrated “significant reductions of the biological half-life of chenodeoxycholic-11, 12-d2 acid, the pool size of chenodeoxycholic acid, and the total bile acid pool size in patients with Crohn’s disease as compared with those in normal subjects” (Nishida et al., 1982). Thus, they concluded the diminished pool size of bile acid in CD was due to impaired absorption at the ileum (Cao et al., 2016). One of the important neglected symptoms of IBD (especially in ileal CD) is BAM (Vítek, 2015). One of the main reasons for BAM in patients with CD is the impaired distal ileum where most of the conjugated and unconjugated bile acids are reabsorbed (Nishida et al., 1982). This statement is also supported by a study in pediatric IBD patients showing BAM in 86% of patients with no or minimal CD clinical activity (Gothe et al., 2014). In 1967, Hofmann defined this phenomenon as “cholerheic enterohepathy,” known as “type 1 or secondary bile acid malabsorption,” which can lead to diarrhea caused by reduced BA reabsorption due to ileal pathology (Hofmann, 1967; Pavlidis et al., 2015). More specifically, BAM in patients with CD involving ileal disease affects colonic fluid and electrolyte secretion leading to diarrhea (Vítek, 2015).

A case-control study (by Uchiyama et al., 2018) study suggested various reasons for BAM in CD patients. In this regard, “mRNA expression levels of ASBT, breast cancer-related protein (BCRP), sulfotransferase family 2A member 1 (SULT2A1), and fibroblast growth factor 19 (FGF-19) were significantly lower in inflamed regions in patients with active Crohn’s ileitis than in controls” (Uchiyama et al., 2018). The reduced level of FGF-19 enhances the hepatic synthesis of bile acid thereby leading to increased intraluminal bile acid concentration and diarrhea in CD patients (Uchiyama et al., 2018). However, the malabsorption of bile acid is so significant that the hepatic synthesis does not atone for the bile acid loss leading to increased concentration of bile acid in the feces.

Interestingly, studies have demonstrated a significant reduction of nuclear receptors including pregnane X (PXR) and farnesoid X (FXR) in CD patient (Wilson et al., 2020). These nuclear receptors regulate pathways that are compromised in patients with IBD. Specifically, bile acids are endogenous ligands for these nuclear receptors. The CD-induced dysregulation of bile acid profiles leads to reduced expression of these receptors, which causes attenuated levels of key target genes (i.e., CYP450 3A4 and FGF-19). Clinically, this would impact optimal dosing of CYP3A4 substrate drugs (e.g., corticosteroids) in patients with CD. Moreover, the reduction in FGF-19 could cause increased bile acid excretion in the feces and IBD-induced diarrhea (Uchiyama et al., 2018; Wilson et al., 2020).

Finally, severe BAM in patients with CD leads to a significant reduction of the intraduodenal bile acid pool, which impairs the micelle formation involved in fat digestion and cholesterol absorption. Further details regarding the BAM-induced alteration of cholesterol metabolism and absorption have been presented previously in the relevant literature (Färkkilä and Miettinen, 1990; McGillicuddy et al., 2009; Romanato et al., 2009; Vítek, 2015). BAM can also lead to many pathophysiological sequelae such as non-alcoholic steatohepatitis (NASH), which are beyond the scope of this review (Appleby et al., 2019).

## Bile Acid Transporters in IBD

A study conducted by Jahnel et al. demonstrated a significant reduction of about 36% in ileal ASBT mRNA expression from CD patients (Jahnel et al., 2014). The ASBT expression was also attenuated in CD patients in remission (Jahnel et al., 2014). Jung et al. observed a significant reduction in ASBT expression in the majority of sixteen CD patients compared with controls (Jung et al., 2004) (Table 2). Interestingly, this study illustrated the activation of human ASBT gene (SLC10A2) by direct binding of dexamethasone and budesonide to the glucocorticoid receptors in the promoter region (Jung et al., 2004). This result implicates an action for glucocorticoids on the human bile acid uptake system, with the possibility of a treatment-related effect on IBD (Jung et al., 2004). Thus, one of the therapeutic effects of glucocorticoids in IBD could be partly explained because of an increased expression of ASBT.

**TABLE 2:** Bile acid transport proteins and bile acid malabsorption in human IBD.

IBD type	Reference number	Effect(s) on BA transporters	Effect(s) on bile acids or lipids
Crohn’s disease and Ulcerative Colitis	20	Decreased ASBT expression (36% reduction of ASBT mRNA expression in active CD and UC and in CD in remission)	Decreased uptake of bile acid, diarrhea
Crohn’s disease	56	Decreased ASBT expression by 7.5%	Not measured

## Bam Diagnosis

One pertinent study demonstrated that the localization of inflammation is not enough to determine the severity of BAM. Therefore, laboratory testing of BAM is needed for all symptomatic patients to identify those candidates for BA sequestrants (e.g., cholestyramine) (Lenicek et al., 2011). “The gold standard diagnosis of BAM is the TauroH-23-(^75^Se) selena-25-homocholic acid 23-seleno-25homo-tauro-cholic acid-test (SeHCAT)” (Lenicek et al., 2011; Fani et al., 2018). In this test, the isotope is orally administered and after 7 days the remaining isotope is measured using a gamma camera. BAM is indicated if the retention of radiolabeled bile acid ^75^SeHCAT is less than 10–15% of the administered tracer (Lenicek et al., 2011).

Other studies have utilized the C4 test to diagnose BAM. In this regard, 7α-hydroxy-4-cholesten-3-one (C4) is a bile acid precursor. For instance, Camilleri et al., demonstrated an increased level of 7αC4 in 46% of patients having ileal disease and 55% in patients with ileal resection (Camilleri et al., 2009). This technique may represent a non-invasive diagnostic advantage to potentially diagnose BAM in patients with ileal disease.

## BAM Treatment-IBD

Type 1 BAM was identified to be secondary to ileal dysfunction, as related to CD in the ileum and/or ileal resection. “The estimated prevalence of BAM is >90% in patients with resected CD and 11–52% for non-resected CD patients” (Barkun et al., 2013). Studies have shown that approximately 83% of chronic diarrhea in patients with BAM has resolved with short-course cholestyramine therapy. “Other bile acid sequestering agents, such as colestipol and colesevelam, are currently being investigated for the treatment of BAM-associated diarrhea” (Barkun et al., 2013).

Using a post hoc analysis comparing colesevelam alone, cholestyramine alone, loperamide alone, and colesevelam with loperamide; the results suggested colesevelam was an effective treatment for post-operative BAM in CD compared to other options (Devarakonda et al., 2019; Valencia-Rodríguez et al., 2019). Hofmann and Poley found that CD patients with <1 m intestinal resections responded well to bile acid sequestrants, while those with >1 m resections did not respond (Hofmann and Poley, 1969). Glucocorticoids may improve BAM in patients with active ileal inflammation by remission induction (Jung et al., 2004; Pattni and Walters, 2009).

## Summary

Many pre-clinical studies have shown BAM in animal models of intestinal inflammation/IBD. Decreased ASBT expression was noted in many of these studies, providing a mechanistic basis for the BAM. BAM related studies in patients with ileal Crohn’s Disease have demonstrated a reduced pool of bile acids, as the ileum is the main site of conjugated and unconjugated bile acid reabsorption. Type 1 BAM is a neglected and underdiagnosed symptom of CD, which has led to many cases of diarrhea due to bile acid spillover into the colon. Like the pre-clinical data, relevant studies have also demonstrated significant reductions of expression for ASBT.

In one clinical study, most patients with BAM associated diarrhea responded to the short-term use of colesevelam. Some studies have shown the efficacy of glucocorticoids in inducing remission and as a potential treatment for CD. Positive steroid treatment results have occurred in a canine model of intestinal inflammation. Future joint studies, which utilize learnings from both pre-clinical and clinical studies, may help to further delineate optimal treatments for BAM-associated diarrhea in patients with CD.

## Author Contributions

Both authors contributed equally to this manuscript.

## Conflict of Interest

The authors declare that the research was conducted in the absence of any commercial or financial relationships that could be construed as a potential conflict of interest.
